# Cost-effectiveness of Methadone Maintenance Treatment in Prevention of HIV Among Drug Users in Shiraz, South of Iran

**DOI:** 10.5812/ircmj.7801

**Published:** 2014-01-05

**Authors:** Ali Keshtkaran, Alireza Mirahmadizadeh, Alireza Heidari, Mehdi Javanbakht

**Affiliations:** 1 School of Management and Medical Information Sciences, Shiraz University of Medical Sciences, Shiraz, IR Iran; 2 Shiraz AIDS Research Center, Shiraz University of Medical Sciences, Shiraz, IR Iran; 3 Health Management and Social Development Research Center, Golestan University of Medical Sciences, Gorgan, IR Iran; 4 Health Economics Research Unit, Institute of Applied Health Sciences, University of Aberdeen, Aberdeen, UK

**Keywords:** Cost-Benefit Analysis, Methadone, Maintenance, Therapeutics, HIV

## Abstract

**Background:**

The increase in high-risk injections and unsafe sexual behaviors has led to increased HIV infection prevalence among Intravenous Drug Users (IDUs). The high costs of HIV/AIDS care and low financial resources necessitate an economic evaluation to make the best decision for the control of HIV/AIDS.

**Objectives:**

This study was conducted to determine the cost-effectiveness of Methadone Maintenance Treatment (MMT) centers in HIV infection prevention among drug users.

**Materials and Methods:**

In this interventional study, we included all the seven MMT centers and the drug users registered there (n = 694). We calculated all the costs imposed on the government, i.e. Provider of case. Mathematical models were used to estimate the number of HIV cases averted from high-risk behaviors. Sensitivity analyses were performed to show the effects of uncertainty in parameters on the number of HIV cases averted and also Incremental Cost-Effectiveness Ratio (ICER).

**Results:**

Based on the averted models, the selected MMT centers could prevent 128 HIV cases during 1 year. The total cost was $ 547423 and that of HIV/AIDS care in the no intervention scenario was estimated $ 14171816. ICER was $ 106382 per HIV case averted. The results of the sensitivity analysis indicated that MMT intervention was cost-effective even in the worst scenario and ICER varied from $ 39149 to $ 290004 per HIV case averted.

**Conclusions:**

With regard to the high prevalence of drug injection among drug users and considering the high effectiveness and cost-effectiveness of MMT centers in preventing HIV infection, establishment of MMT centers in regional and national levels seems reasonable.

## 1. Background

AIDS is the most common deadly infectious disease and is considered as one of the most important mortality causes around the world (1). Unfortunately, there is no treatment or vaccine for HIV/AIDS (2). In Iran, the most common route of HIV transmission is drug injection via shared needle and syringe among IDUs (3); most of them are male, sexually active, and susceptible to transmit the disease not only by shared needles but also by high-risk sexual behaviors (4). Methadone Maintenance Treatment (MMT) is the most effective treatment for drug abuse (5); it reduces the use of injecting drugs and shared syringes (5-8) and helps to prevent HIV (9, 10). As health care costs have increased, researchers have become interested in the economic evaluation of drug abuse. Since the government is responsible for the investment on treatment and reduction of high-risk behaviors among drug users, it is necessary to investigate economic results of this treatment in order to determine the cost-effectiveness of MMT (11). Cost-effectiveness analysis can show whether MMT should remain in the health care programs or administration of Methadone should be changed to justify the costs. Moreover, this analysis can provide health policymaking professionals with some information that can be used to improve such special services (12). The related literature has so far focused on effectiveness of treatment by Methadone more than its cost-effectiveness (11). Most of the studies have investigated the effect of Methadone on decreasing the high-risk behaviors (13-19) and only a few of them have focused on MMT cost-effectiveness. In these studies, the number of HIV infection averted has been used as an effectiveness factor (20, 21).

The research on the effectiveness and cost-effectiveness of MMT has often been conducted in developed countries where the main rout of transmission of HIV/AIDS is sexual contact (22). On the other hand, the common pattern of HIV transmission in Iran is shared injections (3, 4, 23). Currently, extension of MMT centers and development of their services is unknown for decision makers and justifying the cost of the MMT centers requires documentary and scientific proof 

## 2. Objectives

The main objective of this study was to determine the cost-effectiveness of MMT centers to control and prevent HIV/AIDS infection among drug users.

## 3. Materials and Methods

In this uncontrolled interventional study, data were collected by self-reported method in 694 drug users in seven governmental MMT centers in Shiraz, south of Iran. Their high-risk behaviors were compared before and after MMT intervention. We collected data on the costs using government perspective, i.e. the capital and current costs paid by government to MMT centers. We also used a guideline prepared by UNAIDS (United Nations program on HIV/AIDS) to determine the costs of preventing HIV (22, 24). The main costs included expenses of construction, equipment, and vehicles and the current costs included staff wages, consumables, trips, car rental, transportation expenses, current costs of buildings, and personnel costs. We used the annual cost of HIV care to calculate the non-intervention cost using the following formula: the number of averted cases multiplied by the annual cost of care per HIV infection multiplied by the average of lifetime of each case of HIV/AIDS in Iran.

We used HIV infections averted as the measure of effectiveness and measured the costs and effectiveness during a one-year period. To obtain the effectiveness of MMT, we used avert models available in the booklet entitled as "Evaluating Programs for HIV/AIDS Prevention and Care in Developing Countries" published by International Family Health (25). The numbers of HIV infections averted were calculated using a mathematical model designed by Weinstein and colleagues (26). It determines the changes in dug users' possible risky behaviors, revealing the probability of getting or transmitting the infection. This model was offered for sexual contacts and we slightly changed the formula to estimate the HIV infections related to shared injections. The probability of the risk of the studied drug injection to be infected users (A) through the shared injection of other IDUs (B) was calculated by the following equation:

P _B→A_ = 1 - {P_B_ [(1 - ROT) ^n/2^] + (1 - P_B_)} ^m^

In which P is HIV prevalence among IDUs, ROT is the rate of transmission of HIV, n is the average number of shared injections per week and m is the average number of injecting partners in each session. We added all these possibilities and calculated the number of those likely to get infected. We assumed that the position of the individuals under the study changes randomly in every injection session, and totally half of them are at risk of infection; therefore, all the injections were divided by 2 (n/2).

The probability of risk to B from shared injection of A is represented by the following equation:

P _A→B_ = 1 - {P_A_ [(1 - ROT) ^n/2^] + (1 - PA)} ^m^

After determining the probability of infection, we multiplied it by the number of injecting partners and then multiplied it by the probability of being non-HIV; hence, the number of infected people by each individual was calculated. Finally, we subtracted the new estimated HIV infections before and after their arrival to MMT centers. Thus, we estimated HIV infection averted from shared injection in one year. The probability of risk from sexual contact is represented by the following equation:

P _B→A_ = 1 - {P_B_ [1 – ROT _B→A_ (1 - f.e)] ^n^ + (1 - P_B_)} ^m^

P _A→B_ = 1 - {P_A_ [1 – ROT _A→B_ (1 - f.e)] ^n^ + (1 – P_A_)} ^m^

In which P is HIV prevalence among sexually high risk groups, ROT is the rate of HIV transmission by sexual contacts, n is number of sexual acts with each partner and m is the average number of sexual partners. The values of all external parameters, in our models, are shown in Table 1. For some parameters, we chose a value near the middle as the baseline value. To determine the effectiveness of the allotted costs to the sample under the study, the incremental cost-effectiveness ratio was calculated. So, the difference between the intervention and non-intervention costs was divided into the difference of effectiveness (the number of case averted HIV infection) in two states (intervention and non-intervention). One-way sensitivity analysis was conducted to examine the effect of uncertainty of two key parameters, the prevalence of HIV infection among IDUs, and the rate of transmission of HIV infection in needle injection on HIV case averted. Baseline values of possible external parameters and ranges are shown in Table 1. 

**Table 1.** External Parameters Used in the Models

Parameters	Min	Base-Case Value(s)	Max	Source
**Rate of HIV transmission from infected male to female**	0.0001	0.001	0.02	(27-43)
**Rate of HIV transmission from infected female to male**	0.003	0.005	0.0082	(27, 28, 40, 44)
**Rate of HIV transmission from infected male to male (insertive anal intercourse)**	0.0003	0.005	0.032	(27, 39-41, 45, 46)
**Rate of HIV transmission from infected male to male (receptive anal intercourse)**	0.003	0.006	0.009	(27, 39, 40, 44, 45, 47)
**Rate of HIV transmission via shared injection**	0.0029	0.01	0.033	(48-51)
**Condom efficacy**	0.35	0.9	0.95	(52)
**HIV prevalence among Iranian IDUs**	0.012	0.2	0.63	(51)
**HIV prevalence among sex workers in Iran**	-	0.05	-	(51)
**Cost of each HIV positive care per month in Iran ($) **	299	922.4	1236.1	(48)

## 4. Results

Of 694 participants, 92.1% (639) were men. Their mean age was 36.6 ± 9.5 years, and HIV prevalence among them was 42% (Table 2). Before arrival to MMT center, 31.66 new cases of HIV had occurred due to the infection and 123.66 new HIV infections had occurred through transmission of the infection. After arrival to MMT center, six new HIV infections were found due to the infection and 21 HIV occurred through giving the infection. All of the estimated HIV infections due to high-risk injections and sexual contact decreased from 155 HIV infections before to 27 cases after arrival to the centers. Overall, 128 cases were averted (Table 3). 

**Table 2.** Distribution of the Characteristics of the Participants Referred to MMT

Characteristics	No. (%)
**Age, y, n = 694, mean ± SD = 33.6 ± 9.5, Median = 35, Range = 53 (14-67)**	
≤ 20	7 (1)
21-25	65 (9.4)
26-30	138 (19.9)
31-35	150 (21.6)
36-40	134 (19.3)
41-45	64 (9.2)
46-50	65 (9.4)
> 50	71 (10.4)
**Gender, n = 694**	
Male	639 (92.1)
Female	55 (7.9)
**Marital status, n = 694**	
Single	299 (43.1)
Married	259 (37.3)
Divorce	15 (2.2)
Widow	116 (16.7)
Others	5 (0.7)
**Type of drug at first consumption, n = 694**	
Opium	478 (68.9)
Cannabis	135 (19.5)
Heroin	66 (9.5)
Others and mix	15 (2.1)
**HIV and HCV status**	
HIV, n = 369	151 (41)
HCV, n = 358	175 (49)

**Table 3.** Estimated HIV Infections Averted During one-year Period Based on High Risk Behaviors

High Risk Behaviors	Before Referring		After Referring		Total HIV Averted
	Getting Infection	Giving Infection	Getting Infection	Giving Infection	
**Injecting drug**	30.5	121.32	5.35	20.07	126.4
**Sex with men**	0.34	0.74	0.15	0.11	0.83
**Sex with women**	0.82	1.60	0.68	0.89	0.84
**Sum**	31.66	123.66	6.18	21.07	128.07

The total cost of MMT centers was $ 567675 including 23841.8 dollars for the capital costs and 543833.2 dollars for the current costs. These costs reduced to 547423.6 dollars after omitting the cost of drug users who did not participate in this study. The average cost per HIV infection averted was 4276.7 dollars. Health authorities in Iran believe that the cost of each positive HIV person is equal to 922.4 dollars monthly (48). We have considered 10 years as an average of lifetime of each case of HIV/AIDS in Iran. Thus, the cost of non-intervention was calculated to be 14171816.38 dollars. The ICER was calculated as 106382.39 dollars per HIV infection averted. The total estimated cost saving was 13624392.77 dollars. One-way sensitivity analysis, based on the worst and best scenario, showed that changes in numerous parameters had some effects on the results. The results were, especially, highly sensitive to HIV prevalence among IDUs and rate of HIV transmission via injection. ICER changed from 102017.1 dollars per HIV infection averted in low HIV prevalence to 180264.5 dollars per HIV infection averted in high HIV prevalence. In addition, ICER changed from 290004.1 dollars per HIV infection averted in the low rate of HIV transmission via injection to 39149.4 dollars per HIV infection averted in the high rate of HIV transmission. Sensitivity analysis on the cost of care per HIV infection showed that ICER changed from 31605.5 dollars per HIV infection averted in the low cost of care to 144057.5 dollars per HIV infection averted in the high cost of care scenarios. 

## 5. Discussion

This study was conducted to determine whether the activity of MMT centers could be considered as a cost-effectiveness intervention. We used mathematical models in order to estimate HIV infections averted. The results showed that MMT has averted about 128 new cases of HIV and it is a cost-effective HIV prevention. Zaric et al. in USA reported 264 HIV infections averted among the population with a high prevalence of HIV and 40 cases in the population with low prevalence of HIV (53). Masaki et al. reported that in high and low risk areas in China, they averted 3722 and 1960 HIV infection cases during ten years by doing a five year MMT program, respectively (18). In a research by Bayoumi et al. in Canada, 1191 HIV infection cases were averted in a 10-year period (54). Although these differences limit the possibility of comparison between the studies, lower cost-effectiveness in studies conducted in other countries as compared to this study can account for the fact that MMT costs are low and cost of care is high in Iran, which is the same as other countries.

Although most of the studies claim that MMT interventions can be effective and cost-effective, there is a significant difference between the effectiveness of MMT activities. This difference could originate from the difference of the type of harm reduction activities, modelling of calculating case averted, prevalence of HIV infection among high-risk and general population, and frequency of high-risk behaviors among target groups. Most HIV infections averted are caused by decreasing the high-risk injections. It is because the risk of transmission of HIV infection in high-risk injections is more than unprotected sexual contacts (49-51). Moreover, this study showed that the frequency of high-risk sexual contacts in drug users, who are Methadone recipients, was lower than that of the high-risk injections.

Our study showed that MMT interventions, including education, can decrease high-risk sexual behaviors, but this decrease is less than that in high-risk injections. Furthermore, it is possible that sexual contacts increase after MMT intervention due to factors such as poverty, strong dependence on drugs, seeking pleasure, and lack of attention to adverse effects of unprotected sexual behaviors, which finally lead to transmission of HIV virus. Although these are important factor in HIV prevention, there is little information about individual, social and ecologic factors of high-risk sexual behaviors, the role of drug abuse at the beginning and after illegal sexual relationship, and unsafe behaviors following it (52, 55). This study was performed from provider perspective (government) for two reasons: first, Iranian government provides budget of HIV/AIDS and MMT care centers and second calculation of the cost from social perspective is so hard and impossible. Therefore, in our study, performing MMT saved 106382.3 dollars per HIV infection averted and reduced the government costs. In a study by Masaki et al., the cost was estimated 2686 dollars due to MMT in the high-risk areas and it was 5101 dollars in the low-risk areas (20). In Holtgrave and Pinkerton's study (1997), the cost per HIV infection averted was 4000 dollars (19). Bayoumi et al. (2008) in Canada estimated 20100 dollars as the cost per HIV infection averted (54). Likewise, the cost per HIV infection averted was 359 dollars in the study by Kumaranyake et al. (21).

Some of the limitations of this study included difficulties in attracting the drug users' participation, probable inability to recall some information in self-reporting method, and lack of precise report of the sexual information by the drug users in the interview. Our data were directly gathered from the participants through an interviewer-administered questionnaire. A previous study had demonstrated that IDUs might under-report stigmatized behaviors, such as needle sharing and especially sexual behaviors. Nevertheless, self-report of sexual and a drug use behavior in IDUs has been demonstrated to have acceptable reliability and validity (55). If we had increased the duration of our study to more than one year and had used Markov function or decision tree models, it could have led to more desirable and reliable results (54, 55). The simultaneous use of two-way sensitivity analysis on all of the effective parameters on costs and outcomes can be useful as well. Finally, concerning the high effectiveness and cost-effectiveness of MMT centers in preventing HIV, it is necessary to develop these centers regionally and nationally in Iran to cover the high-risk people.
